# Construction and Comparison of ceRNA Regulatory Network for Different Age Female Breast Cancer

**DOI:** 10.3389/fgene.2021.603544

**Published:** 2021-04-21

**Authors:** Zhi-Qin Liu, Gao-Tao Zhang, Li Jiang, Chun-Qing Li, Que-Ting Chen, Du-Qiang Luo

**Affiliations:** ^1^Key Laboratory of Pharmaceutical Quality Control of Hebei Province, Institute of Life Science and Green Development, College of Pharmaceutical Science, Hebei University, Baoding, China; ^2^Key Laboratory of Medicinal Chemistry and Molecular Diagnosis of Ministry of Education, College of Life Science, Hebei University, Baoding, China; ^3^Department of Breast Surgery, Affiliated Hospital of Hebei University, Hebei University, Baoding, China

**Keywords:** age, breast cancer, ceRNA network, lncRNA, miRNA

## Abstract

Studies have shown the difference appearing among the prognosis of patients in different age groups. However, the molecular mechanism implicated in this disparity have not been elaborated. In this study, expression profiles of female breast cancer (BRCA) associated mRNAs, lncRNAs and miRNAs were downloaded from the TCGA database. The sample were manually classified into three groups according to their age at initial pathological diagnosis: young (age ≤ 39 years), elderly (age ≥ 65 years), and intermediate (age 40–64 years). lncRNA-miRNA-mRNA competitive endogenous RNA (ceRNA) network was respectively constructed for different age BRCA. Then, the biological functions of differentially expressed mRNAs (DEmRNAs) in ceRNA network were further investigated by Gene Ontology (GO) and Kyoto Encyclopedia of Genes and Genomes (KEGG) pathway analysis. Finally, survival analysis was used to identify prognostic biomarkers for different age BRCA patients. We identified 13 RNAs, 38 RNAs and 40 RNAs specific to patients aged ≤ 39 years, aged 40–64 years, and aged ≥ 65 years, respectively. Furthermore, the unique pathways were mainly enriched in cytokine-cytokine receptor interaction in patients aged 40–64 years, and were mainly enriched in TGF-beta signaling pathway in patients aged ≥ 65 years. According to the survival analysis, AGAP11, has-mir-301b, and OSR1 were respectively functioned as prognostic biomarkers in young, intermediate, and elderly group. In summary, our study identified the differences in the ceRNA regulatory networks and provides an effective bioinformatics basis for further understanding of the pathogenesis and predicting outcomes for different age BRCA.

## Introduction

Breast cancer (BRCA) is one of the common cancers in women worldwide, and its incidence is increasing year by year. Some studies have demonstrated that the younger and older BRCA patients had a poorer prognosis compared with the intermediate age patients (Brandt et al., 2015). However, the molecular mechanism implicated in this disparity have not been elaborated. Thus, it is crucial to explore molecular pathogenesis and intracellular signaling pathways, and identify new diagnosis, therapy, and prognosis indicator for different age BRCA patients. A large number of studies have demonstrated that competitive endogenous RNAs (ceRNAs) perform important roles in the genesis, development, and prognosis of BRCA (Qi et al., 2015). The ceRNA hypothesis proposed by Salmena et al. (2011), a new regulatory mechanism between coding mRNA and non-coding RNA (ncRNA), affect mRNA functions at the posttranscriptional level. The lncRNAs sever as ceRNAs to communicate with mRNAs by competitively binding to miRNAs. Many ceRNAs act as main regulators that disrupt the expression levels of target genes (Ergun and Oztuzcu, 2015). Some research have shown that lncRNA–miRNA–mRNA ceRNA network implicated in the progression and prognosis of BRCA (Fan et al., 2018; Naorem et al., 2020). However, the lncRNA–miRNA–mRNA ceRNA network of different age BRCA have not yet been explored as so far.

In this study, BRCA expression data of lncRNA, miRNA, and mRNA from TCGA database and their corresponding clinical data were collected. The sample were manually classified into three groups according to their age at initial pathological diagnosis: young, elderly and intermediate. After the analysis of differentially expressed genes, we compared the ceRNA networks, related enrichment analysis results, and key RNAs associated with overall survival time for three age groups BRCA. Finally, we found that some unique RNAs which were tightly related to different age BRCA. Based upon this research, we can not only further understand the molecular mechanism but also provide potential diagnosis, treatment and prognosis biomarkers for different age BRCA. Our results demonstrated that age increased biological complexity of BRCA, and it is a factor that must be considered in the diagnosis, treatment, and prognosis of BRCA patients.

## Materials and Methods

### Data Collection and Processing

The raw mRNA, lncRNA, and miRNA expression data and clinical information of BRCA were acquired from TCGA Data Portal^1^. Among them, mRNA and lncRNA sequencing data were derived from 1,208 samples, including 112 normal samples and 1,096 BRCA samples, and miRNA expression data from 1,193 samples, including 103 normal samples and 1,090 BRCA samples. The sample were manually classified into three groups according to patients age at initial pathological diagnosis: young (age ≤ 39 years), elderly (age ≥ 65 years), and intermediate (age 40–64 years). The cutoff points were set based on other historical studies (Kan et al., 2018; Ma et al., 2020). In young group, mRNA and lncRNA sequencing data were derived from 86 samples, including nine normal samples and 77 BRCA samples, and miRNA expression data from 83 samples, including eight normal samples and 75 BRCA samples. In elderly group, mRNA and lncRNA sequencing data were derived from 369 samples, including 31 normal samples and 338 BRCA samples, and miRNA expression data from 367 samples, including 29 normal samples and 338 BRCA samples. In intermediate group, mRNA and lncRNA sequencing data were derived from 753 samples, including 72 normal samples and 681 BRCA samples, and miRNA expression data from 743 samples, including 66 normal samples and 677 BRCA samples. The relevant data acquired from TCGA are publicly available and open-ended, and therefore approval of the ethics committee was not required.

### Identification of Differentially Expressed mRNAs, miRNAs, and lncRNAs

The downloaded data were normalized, and differentially expressed lncRNAs, miRNAs, and mRNAs in the BRCA and normal tissue samples of different age women by using the R software and edgeR Bioconductor package. The screening criteria of the three kinds of dysregulated RNAs were as follows: (a) absolute log_2_folds change (log_2_FC) > 2; (b) adjusted *P*-value < 0.01. Corresponding heat maps and clustering were generated using the gplots R package.

### Prediction of lncRNA–miRNA and miRNA–mRNA Interactions

The lncRNA–miRNA interaction was predicted by using miRcode database^2^, and the differentially expressed lncRNAs (DElncRNAs) and the differentially expressed miRNAs (DEmiRNAs) which could be compared successfully were obtained. Furthermore, TargetScan^3^, miRDB^4^, and miRTarBase^5^ were used to predict the target mRNAs of DEmiRNAs. Genes exist in all three databases were considered as target genes of these DEmiRNAs. To further enhance the credibility of bioinformatics analysis, intersection between target mRNAs of miRNAs and differentially expressed mRNA in BRCA was obtained by using Venny diagram analysis, which was further analyzed as the differentially expressed mRNA (DEmRNA). In the end, the matched DElncRNA–DEmiRNA and DEmiRNA–DEmRNA pairs were acquired.

### Construction of the DElncRNA–DEmiRNA–DEmRNA ceRNA Network

Based on the obtained DElncRNA–DEmiRNA and DEmiRNA–DEmRNA pairs, the ceRNA network of BRCA at different age groups were reconstructed, which was visualized *via* Cytoscape v3.7.1 software. Furthermore, the cytoHubba plugin was used to identify the top 20 genes in the network based on closeness.

### Gene Ontology and KEGG Pathway Analysis

To explore the different potential biological processes of dysregulated mRNAs in the ceRNA network of BRCA. Gene Ontology (GO) Biological Process enrichment analysis and Kyoto Encyclopedia of Genes and Genomes (KEGG) pathway enrichment analysis was performed using clusterProfiler R package. GO and KEGG enrichment analyses were based on the threshold of *P* < 0.05. Finally, we compared the enrichment results and displayed the results using ggplot2 and GO plot R packages.

### Overall Survival Analysis

Setting the median as the screening standard and according to the cancer expression data of every RNA in the ceRNA network, we respectively classified the young (age ≤ 39 years), elderly (age ≥ 65 years), and intermediate (age 40–64 years) samples into two groups: high expression or low expression. Combined with the survival time data for BRCA in TCGA, the survival R package (version 3.4.3) was used to analyze the specific lncRNA, miRNA, and mRNA associated with survival. *P* < 0.05 were considered statistically significant.

### Univariate and Multiple Cox Regression Analysis

In order to better evaluate the prognostic value every RNA which has statistical significance in overall survival analysis in the ceRNA network, clinicopathological characteristics (including histological type, clinical stage, lymph node status, ER status, PR status, and Her2 status) was collected, and the univariate Cox regression and multiple Cox regression analysis was adopted. *P* < 0.05 were considered statistically significant.

## Results

### Data Set Acquisition and Identification of Deregulated RNAs

Using | log_2_FC| > 2 and *P*-value < 0.01 as screening cutoff conditions, we calculated mRNA, miRNA, and lncRNA expression profiles between BRCA samples and normal samples at different age groups obtained from TCGA. In group aged ≤ 39 years, there were 200 upregulated lncRNAs, 203 downregulated lncRNAs; 33 upregulated miRNAs, 20 downregulated miRNAs; 565 upregulated mRNAs, 552 downregulated mRNAs. In group aged 40–64 years, there were 830 upregulated lncRNAs, 220 downregulated lncRNAs; 66 upregulated miRNAs, 20 downregulated miRNAs; 1,359 upregulated mRNAs, 764 downregulated mRNAs. In group aged ≥ 65 years, there were 775 upregulated lncRNAs, 280 downregulated lncRNAs; 86 upregulated miRNAs, 15 downregulated miRNAs;1,410 upregulated mRNAs, 740 downregulated mRNAs. The heat maps and volcano plots of lncRNAs, miRNAs, and mRNAs show the differences between BRCA samples and normal samples at different age groups (Supplementary Figures 1–3). We identified 245 common deregulated ncRNAs (26 miRNAs, 219 lncRNAs, Supplementary Figure 4), 126 ncRNAs (18 miRNAs, and 108 lncRNAs) specific to BRCA aged ≤ 39 years, 347 ncRNAs (330 lncRNAs, 17 miRNAs) specific to BRCA aged 40–64 years, and 352 ncRNAs (321 lncRNAs, 31 miRNAs) specific to BRCA aged ≥ 65 years.

### ceRNA Network Construction and Analysis

**FIGURE 1:**
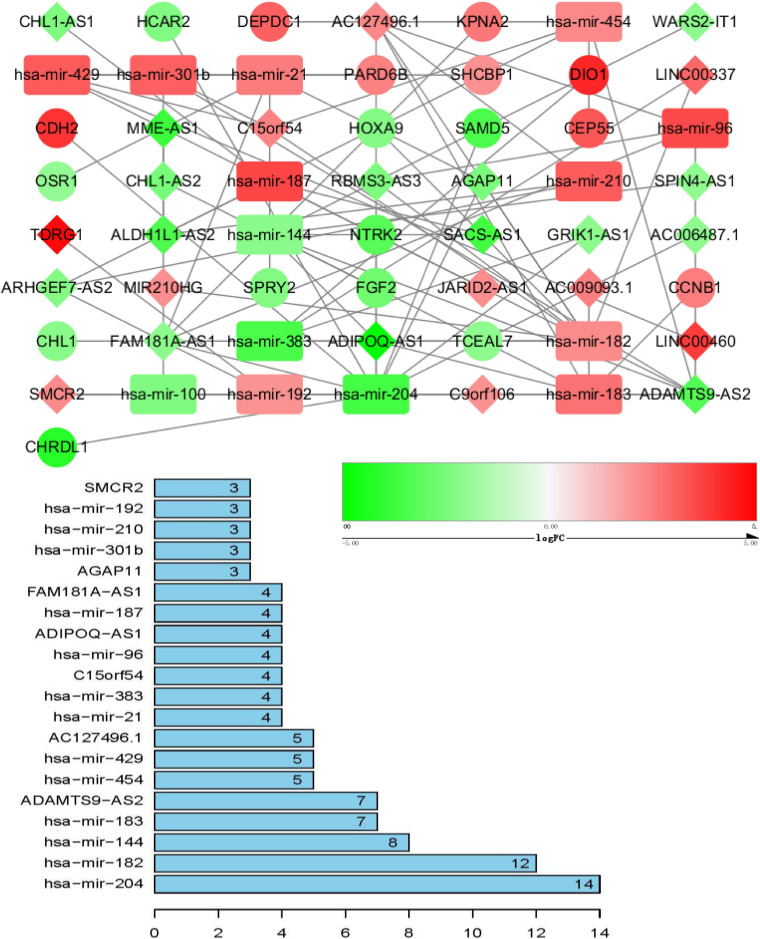
ceRNA networks of BRCA patients aged ≤ 39 years. Red represents upregulation, and green represents downregulation. miRNAs, LncRNAs, and mRNAs in the networks are represented as round rectangles, diamonds, and circles, respectively. Bar plots show the key genes that have the top interaction numbers in whole networks.

**FIGURE 2:**
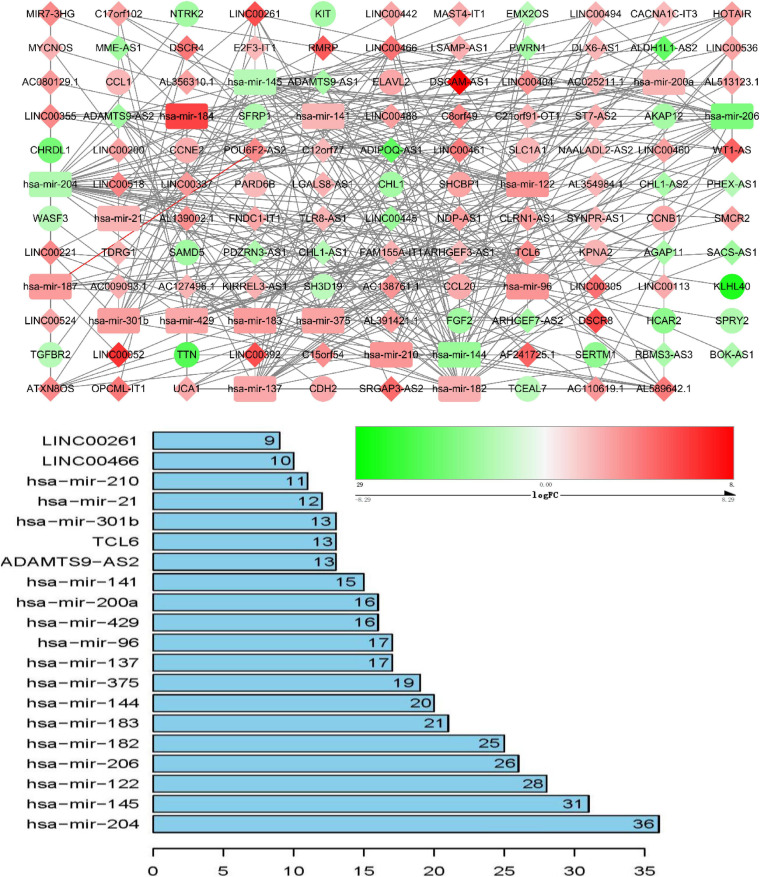
ceRNA networks of BRCA patients aged 40–64 years. Red represents upregulation, and green represents downregulation. miRNAs, LncRNAs, and mRNAs in the networks are represented as round rectangles, diamonds, and circles, respectively. Bar plots show the key genes that have the top interaction numbers in whole networks.

**FIGURE 3:**
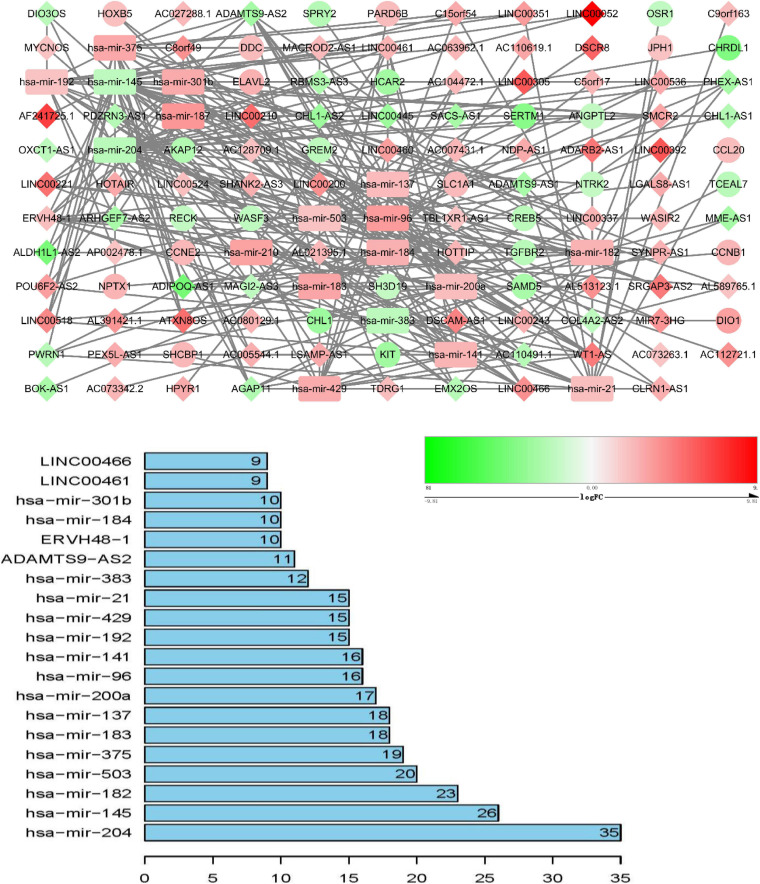
ceRNA networks of BRCA patients aged ≥ 65 years. Red represents upregulation, and green represents downregulation. miRNAs, LncRNAs, and mRNAs in the networks are represented as round rectangles, diamonds, and circles, respectively. Bar plots show the key genes that have the top interaction numbers in whole networks.

ceRNA networks of at different age BRCA were constructed and are presented in Figures 1–3. For a better understanding of the regulatory mechanism differences at different age groups, based on the plug-in cytoHubba in Cytoscape we compared the interactions and RNAs of three age groups. We identified 34 common network RNAs (10 mRNAs, nine miRNAs, 15 lncRNAs, Supplementary Figure 5), 13 RNAs (eight lncRNAs, two miRNAs, and three mRNAs) specific to BRCA aged ≤ 39 years, 38 RNAs (32 lncRNAs, two miRNAs, and four mRNAs) specific to BRCA aged 40–64 years, and 40 RNAs (31 lncRNAs, one miRNAs, and eight mRNAs) specific to BRCA aged ≥ 65 years (Supplementary Tables 1–3). After counting the interaction numbers of each RNA, we found the top three RNAs in the three networks. For BRCA aged ≤ 39 years ceRNA network, they were hsa−mir−204, hsa−mir−182, and hsa−mir−144. For patients aged 40–64 years network, the top three RNAs were hsa−mir−204, hsa−mir−145, and hsa−mir−122. For BRCA aged ≥ 65 years network, the top three RNAs were hsa−mir−204, hsa−mir−145, and hsa−mir−182. We assumed them as hub RNAs which may play important roles in the different aged BRCA network. Bar plots are shown in Figures 1–3.

### Function Annotation of ceRNA Network

**FIGURE 4:**
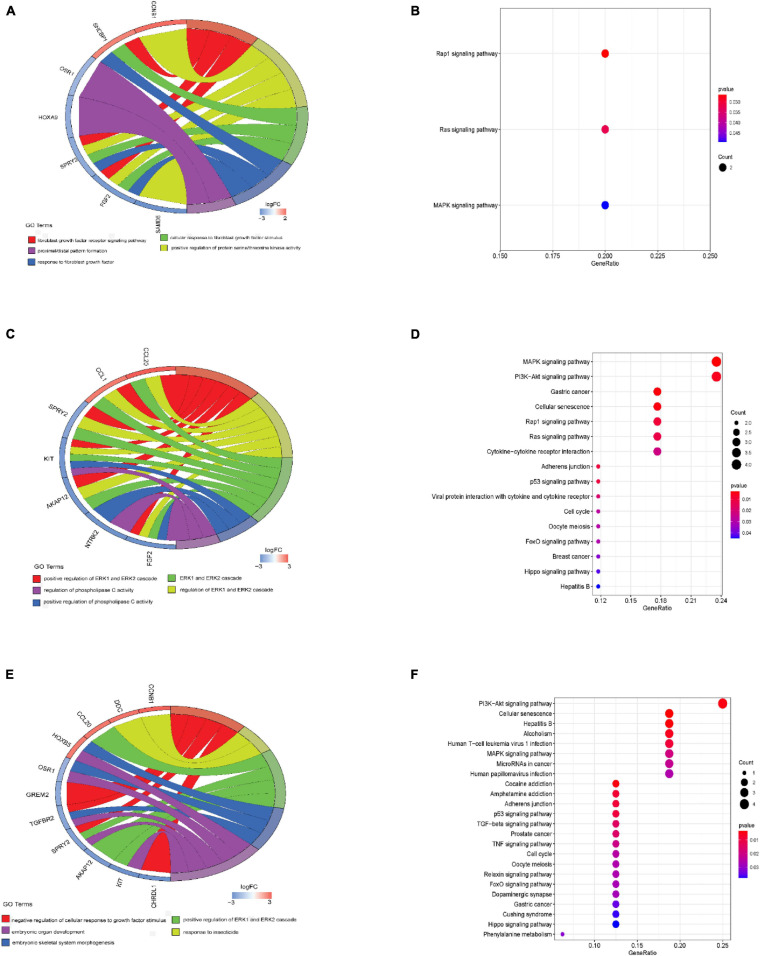
GO biological process and KEGG analyses of DEmRNAs involved in the ceRNA networks. **(A)** GO biological process analysis of BRCA patients aged ≤ 39 years; **(B)** Results of KEGG analysis of BRCA patients aged ≤ 39 years; **(C)** GO biological process analysis of BRCA patients aged 40–64 years; **(D)** Results of KEGG analysis of BRCA patients aged 40–64 years; **(E)** GO biological process analysis of BRCA patients aged ≥ 65 years; **(F)** Results of KEGG analysis of BRCA patients aged ≥ 65 years.

Under the threshold of *P* < 0.05, different age BRCA DEmRNAs had 127 common GO biological process (BP) enrichment terms. For BRCA patients aged ≤ 39 years, the unique BP terms enriched in cancer-related functions were mainly endothelial cell activation, mitotic cytokinetic process, and hyaluronan metabolic process. For patients aged 40–64 years, the unique cancer-related BP terms were mainly related to cellular response to tumor necrosis factor, protein kinase A signaling, peptidyl-tyrosine phosphorylation, and regulation of phosphatidylinositol 3-kinase activity. For patients aged ≥ 65 years, the unique cancer-related BP terms were mainly negative regulation of metalloendopeptidase activity and extracellular regulation of signal transduction. According to KEGG pathway analysis, the common cancer-related pathways of different age BRCA ceRNA network were MAPK signaling pathway. For patients aged 40–64 years, the unique KEGG terms were mainly cytokine-cytokine receptor interaction. The unique pathways of the patients aged ≥ 65 years ceRNA network included TGF-beta signaling pathway, TNF signaling pathway, and MicroRNAs in cancer. These enrichment results are shown in Figure 4 and Tables 1,2.

**TABLE 1:** The main five unique GO biological processes of DEmRNAs involved in the ceRNA networks of different age BRCA patients.

**Group**	**ID**	**Description**	***P* value**
Age ≤ 39 years	GO:0042118	Endothelial cell activation	0.011511585
	GO:0060216	Definitive hemopoiesis	0.019116321
	GO:1902410	Mitotic cytokinetic process	0.021953825
	GO:0010769	Regulation of cell morphogenesis involved in Differentiation	0.033439692
	GO:0030212	Hyaluronan metabolic process	0.035093312
Age 40–64 years	GO:0050708	Regulation of protein secretion	0.000428592
	GO:0071356	Cellular response to tumor necrosis factor	0.000659382
	GO:0010737	Protein kinase A signaling	0.000958848
	GO:0018108	Peptidyl-tyrosine phosphorylation	0.001497798
	GO:0043551	Regulation of phosphatidylinositol 3-kinase activity	0.002646505
age ≥ 65 years	GO:0018958	Phenol-containing compound metabolic process	0.01110598
	GO:1904684	Negative regulation of metalloendopeptidase activity	0.01542851
	GO:0042428	Serotonin metabolic process	0.01542851
	GO:1900115	Extracellular regulation of signal transduction	0.016958658
	GO:1901160	Primary amino compound metabolic process	0.02001207

**TABLE 2:** The common and unique KEGG pathway of DEmRNAs involved in the ceRNA networks of different age BRCA patients.

	**ID**	**Description**	***P* value**
Common	hsa04010	MAPK signaling pathway	
Age 40–64 years	hsa04061	Viral protein interaction with cytokine and cytokine receptor	0.018415834
	hsa04060	Cytokine-cytokine receptor interaction	0.022444793
	hsa05224	Breast cancer	0.037674483
Age ≥ 65 years	hsa05030	Cocaine addiction	0.004127995
	hsa05034	Alcoholism	0.005532436
	hsa05031	Amphetamine addiction	0.00804652
	hsa05166	Human T-cell leukemia virus 1 infection	0.008567374
	hsa04350	TGF-beta signaling pathway	0.014565067
	hsa05215	Prostate cancer	0.01546109
	hsa04668	TNF signaling pathway	0.020287074
	hsa05206	MicroRNAs in cancer	0.021840761
	hsa05165	Human papillomavirus infection	0.025931851
	hsa04926	Relaxin signaling pathway	0.026421972
	hsa04728	Dopaminergic synapse	0.027574614
	hsa00360	Phenylalanine metabolism	0.031371877
	hsa04934	Cushing syndrome	0.037069433

### Overall Survival Analysis of RNAs in the ceRNA Network

**FIGURE 5:**
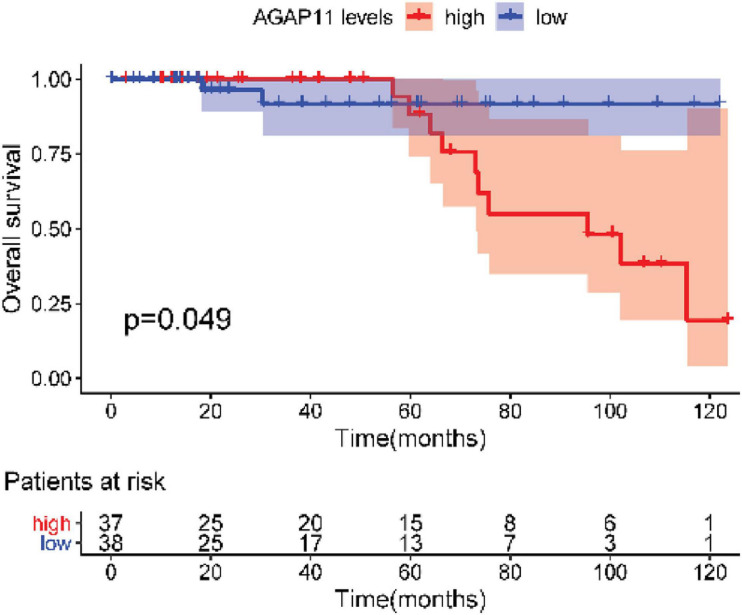
Overall survival analysis of RNAs in the ceRNA network of BRCA patients aged ≤ 39 years.

**FIGURE 6:**
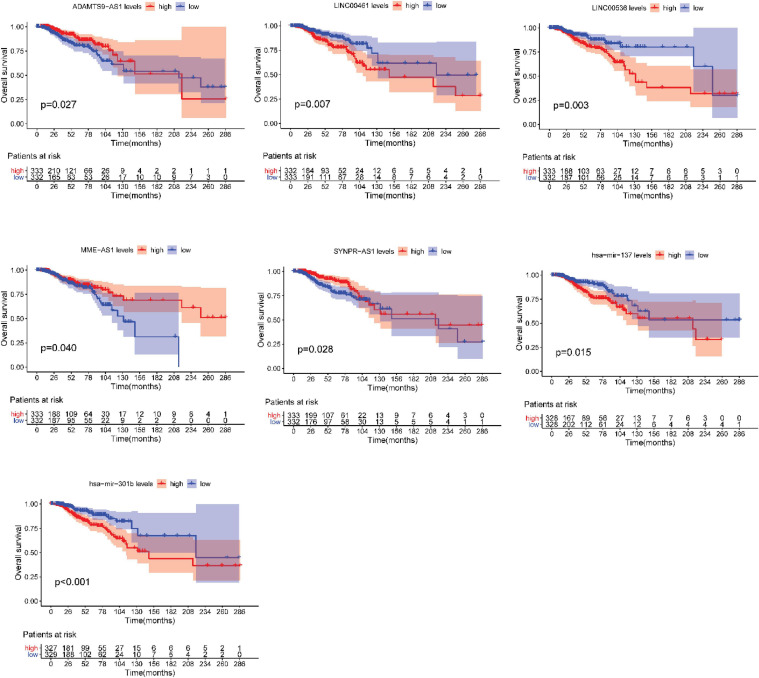
Overall survival analysis of RNAs in the ceRNA network of BRCA patients aged 40–64 years.

**FIGURE 7:**
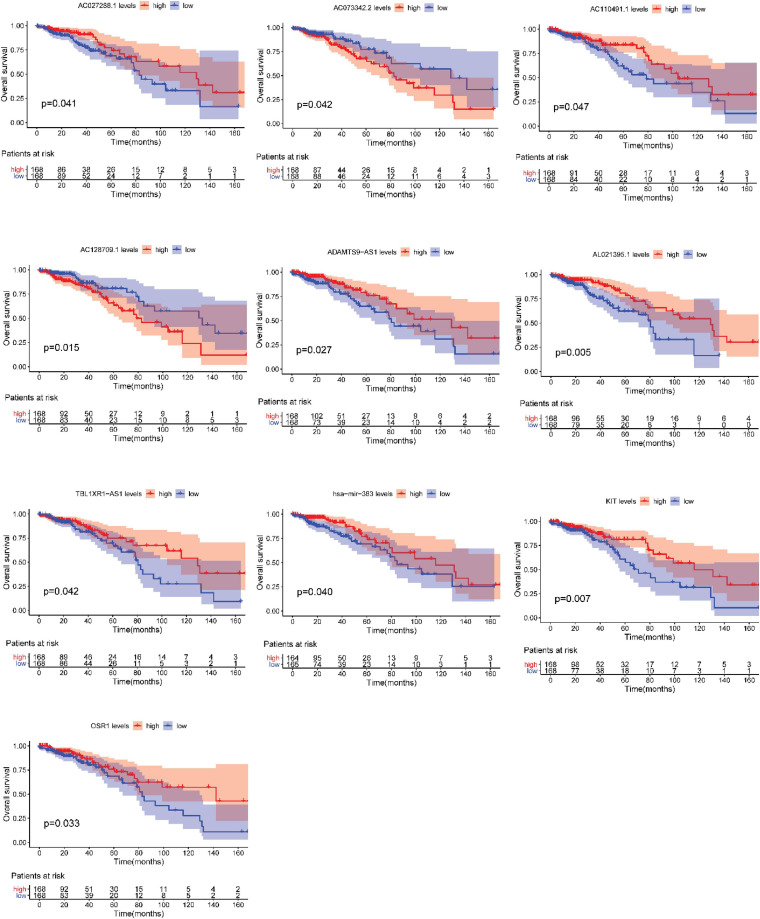
Overall survival analysis of RNAs in the ceRNA network of BRCA patients aged ≥ 65 years.

After testing all of the RNAs in the ceRNA network, KM analysis was performed to investigate the association between DERNAs of the ceRNA network and the prognosis of BRCA patients, and survival curves of different age groups BRCA patients were obtained. For patients aged ≤ 39 years, only one lncRNA, AGAP11 was the relevant RNAs associated with survival time (*P* < 0.05) (Figure 5). For patients aged 40–64 years, five lncRNAs (ADAMTS9-AS1, LINC00461, LINC00536, SYNPR-AS1, and MME-AS1), and two miRNAs (has-mir-137 and has-mir-301b) were the relevant RNAs associated with survival time (*P* < 0.05) (Figure 6). For patients aged ≥ 65 years, seven lncRNAs (AC027288.1, AC073342.2, AC110491.1, AC128709.1, ADAMTS9-AS1, AL021395.1, and TBL1XR1-AS1), two mRNAs (KIT and OSR1), and one miRNA (hsa-mir-383) were the relevant RNAs associated with survival time (*P* < 0.05) (Figure 7).

### Univariate and Multiple Cox Regression Analysis

**FIGURE 8:**
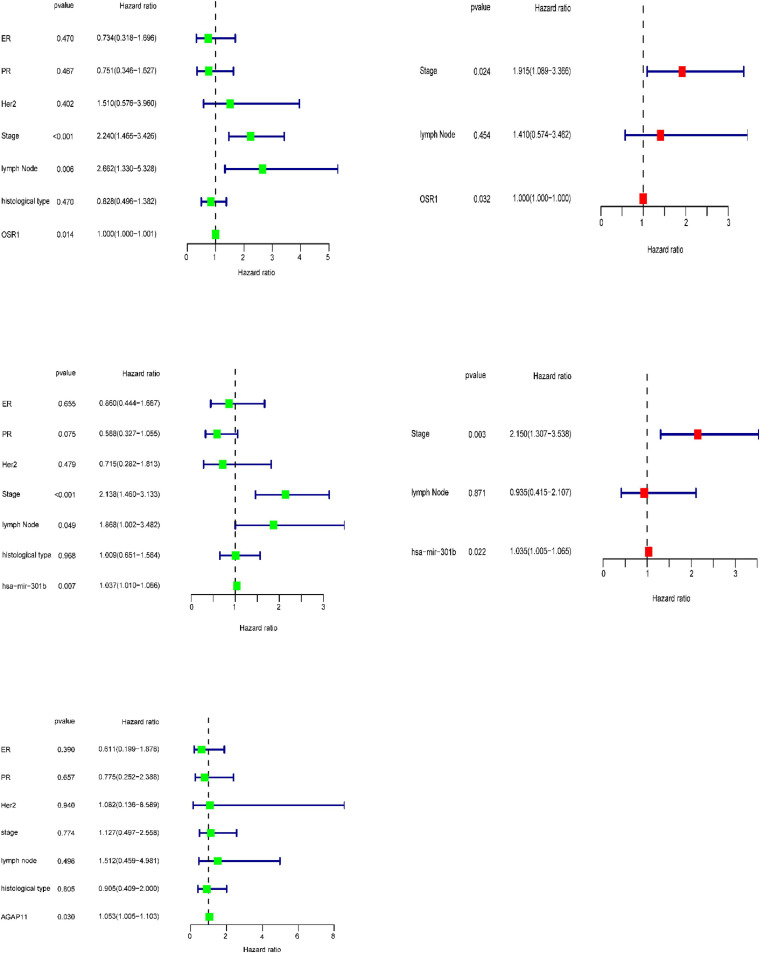
Independent prognostic analysis of RNAs in the ceRNA network of BRCA patients in three age groups.

After performing KM analysis, clinicopathological characteristics (including histological type, clinical stage, lymph node status, ER status, PR status, and Her2 status, Supplementary Table 4) was considered, and every RNA which has statistical significance in overall survival analysis of each age group was subjected to the univariate Cox regression and multiple Cox regression analysis. For patients aged ≤ 39 years, AGAP11 was an independent prognostic factors (Figure 8). For patients aged 40–64 years, has-mir-301b has statistical differences both in univariate Cox regression and in multiple Cox regression analysis (Figure 8). For patients aged ≥ 65 years, OSR1 was an independent prognostic factors (Figure 8).

## Discussion

**FIGURE 9:**
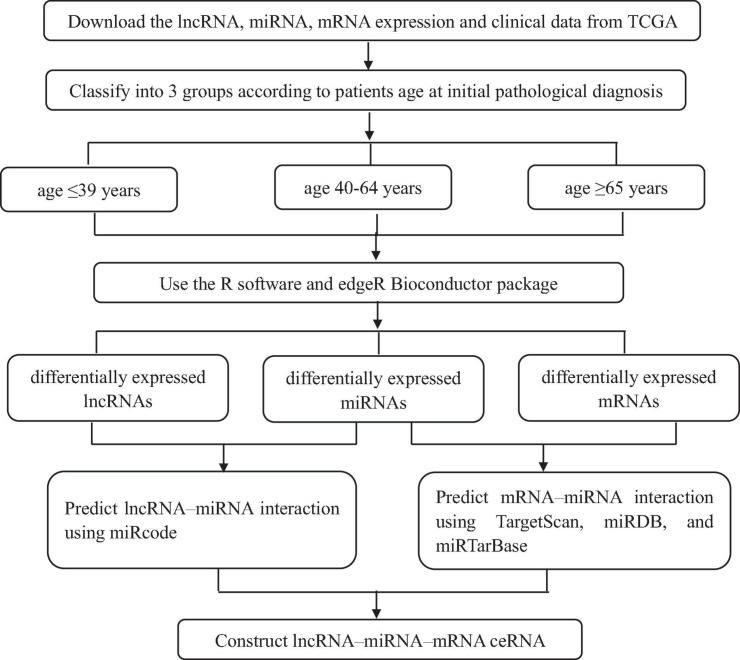
The flow diagram of this study.

Breast cancer is one of the most prevalent malignant tumor and the main causes of cancer death among female worldwide. Studies have shown the difference appearing among the prognosis of patients in different age groups (El Chediak et al., 2017). However, the molecular mechanism implicated in this discrepancy have not been fully elaborated. The ceRNA networks is one of the useful tool for confirming unique biomarkers for early diagnosis, therapy, and prognosis for all kinds of tumors; however, the construction of ceRNA networks among different age BRCA has not been previously performed. This study aimed to construct the ceRNA network for different age BRCA patients, which provides valuable information to further explore the molecular mechanism underlying tumorigenesis and development of disparate age BRCA. Our whole workflow is shown in Figure 9. In our research, there were 403 lncRNAs, 53 miRNAs, and 1,117 mRNAs in BRCA aged ≤ 39 years; 1,050 lncRNAs, 86 miRNAs, and 2,123 mRNAs in BRCA aged 40–64 years; 1,055 lncRNAs, 101 miRNAs, and 2,150 mRNAs in BRCA aged ≥ 65 years with differentially expressed profiles were identified. The numbers of the three kinds of differentially expressed RNAs were smaller in young BRCA than in middle-aged and elderly BRCA, which revealed that young BRCA patients has less heterogeneity.

After constructing the lncRNA–miRNA–mRNA ceRNA networks of different age BRCA, we compared the three networks, and found 34 common RNAs across different age group ceRNA networks which played specific roles in each age group through competing with diverse mRNAs. Among the 34 common RNAs, hsa-mir-204, hsa-mir-182, and hsa-mir-183 had more interactions than other RNAs. Through consulting relevant literature, we found that hsa-mir-204, hsa-mir-182, and hsa-mir-183 has been studied to some extent in the field of BRCA. miR-204 took part in the regulation of proliferation, invasion, metastasis, and apoptosis of BRCA cells *via* immediately controlling FOXA1 (Shen et al., 2017). MiR-182 regulated the growth, invasion, and the HIF-1α-VEGF-A axis of BRCA cells *via* manipulating FBXW7 (Chiang et al., 2016). The miR-183 overexpression were related to a negative consequence among women BRCA (Marino et al., 2014). In present study, down-regulated hsa-mir-204 and up-regulated hsa-mir-182 and hsa-mir-183 not only had involved with ceRNA network but also had more interactions than other RNAs in three age groups. These results illustrated that hsa-mir-204, hsa-mir-182, and hsa-mir-183 regulation are crucial for the development of all ages female patients with BRCA, which were in line with most of the previous studies (Li et al., 2014; Wang K. et al., 2019).

In order to explore the unique diagnostic and prognostic indicators, as well as therapeutic targets of different age BRCA, we mainly focus on the specific RNAs of each age group. In BRCA aged ≤ 39 years ceRNA network, hsa-mir-454 and FAM181A-AS1 not only have specificity but also had more interactions. After consulting the relevant documents, we found that hsa-mir-454 played a non-negligible role in the development and prognosis of multiple tumor. For example, miR-454 restrained the growth and invasion of ovarian cancer cells *via* regulating E2F6 (An et al., 2020). The miR-454-3p may be a meaningfully standalone prognosis marker for hepatocellular carcinoma (Li et al., 2019). Zuo et al. (2019) demonstrated that hsa-miR-454 inhibited glioblastoma through down-regulating NFATc2 expression. Song identified that miR-454-3p accelerated the growth of cervical cancer *via* manipulating TRIM3 (Song et al., 2019). About the function of hsa-mir-454 in BRCA, one study found that MiR-454-3p may be used as diagnosis and treatment biomarker for BRCA metastasis patients (Ren et al., 2019). In present study, down-regulated FAM181A-AS1 had more interaction level. However, after reviewing the relevant literature, we found that there are few studies about FAM181A-AS1. Considering its unique role in patients aged ≤ 39 years ceRNA network, we speculated that FAM181A-AS1 weighed heavily in the development of BRCA aged ≤ 39 years. Therefore, the special function of FAM181A-AS1 is needed to be further investigated for BRCA patients aged ≤ 39 years.

In BRCA aged 40–64 years network, hsa-mir-206, hsa-mir-122, TCL6, and LINC00261 have specificity in the top twenty RNAs with more interactions. *Via* reviewing the relevant literature, we found that the role of hsa-mir-206 and hsa-mir-122 in BRCA had been extensively investigated in recent years. For example, Samaeekia et al. (2017) demonstrated that miR-206 played a crucial role in repressing stemness and metastasis of BRCA through regulating MKL1/IL11 signal passage. Fu et al. (2015) revealed that Hsa-miR-206 exerted the inhibition of growth and aggression in BRCA cells through adjusting Cx43. Wang et al. (2014) reported that miR-206 may be served as a potential metastatic marker for triple-negative BRCA. One study found that miR-122-5p inhibited CHMP3 expression *via* MAPK signaling pathway, which accelerated invasion and epithelial-mesenchymal transition in triple-negative BRCA (Wang and Wang, 2020). miR-122 repressed the growth of BRCA cells through manipulating PI3K/Akt/mTOR/p70S6K signaling pathway (Wang et al., 2012). Although the function of hsa-mir-206 and hsa-mir-122 in BRCA attract widespread attention, however, we found that they are exclusive for BRCA aged 40–64 years ceRNA network in present study. Therefore, we assumed that hsa-mir-206 and hsa-mir-122 played crucial role in the development of BRCA patients aged 40–64 years, which need to be further explored. Recently, T cell leukemia/lymphoma 6 (TCL6), a less-studied lncRNA has been identified as a diagnosis and prognostic marker in some cancer, such as clear cell renal cell carcinoma, hepatocellular carcinoma (Liu et al., 2020; Luo et al., 2020). About the function of TCL in BRCA, one study revealed that TCL6 was associated with immune infiltration and identified as a poor prognosis predicator in BRCA patients (Zhang Y. et al., 2020). LINC00261 has an inhibitory effect on several kinds of cancer, and was identified as a prognostic indicator guided clinical practice, such as lung cancer, pancreatic cancer (Guo C. et al., 2020; Wang X. et al., 2020). Guo G. et al. (2020) demonstrated that LINC00261 was downregulated in BRCA tissues compared with adjacent normal tissues. However, in present study, we found that LINC00261 was upregulated in the cancer tissues for aged 40–64 years BRCA patients. This contradiction may be related with inclusion criteria of patients, and need to be further explored in the future.

Among the unique RNAs of BRCA aged ≥ 65 years network, hsa-mir-503 and ERVH48-1 have more interaction levels. By consulting the pertinent literature, we found that the function of hsa-mir-503 in BRCA have been extensively investigated in recent years. Zhao et al. (2017) demonstrated that miR-503-3p induced epithelial-mesenchymal transition in BRCA *via* immediately manipulating SMAD2 and E-cadherin. Yan et al. (2017) reported that mir-503/IGF-1R pathway play important role in controlling the growth and aggression of BRCA cells. Another study showed that mir-503 repressed the BRCA cells vitality through regulating CCND1 (Long et al., 2015). Although the vital role of hsa-mir-503 in BRCA was identified by these literature, the mir-503 dysregulation was only found in BRCA aged ≥ 65 years network in our study. Therefore, we presumed that hsa-mir-503 may be play crucial role in the pathogenesis of BRCA patients aged ≥ 65 years. ERVH48-1, an endogenous retroviral provirus, plays a role in tumor formation. Zhang et al. (2019) demonstrated that lncRNA ERVH48-1 may be act as a latent signature of prognosis and treatment for tongue squamous cell carcinoma patients. Qi et al. (2019) confirmed ERVH48-1 as a latent prognostic element for lung squamous cell carcinoma patients based on the ceRNA network analysis. Our study is the first report of the key role of ERVH48-1 in ceRNA networks, and its dysregulation is crucial for the development of BRCA aged ≥ 65 years. The special function of ERVH48-1 in BRCA still needs to be further explored.

To explore the potential biological functions of DEmRNAs involved in ceRNA network in different age BRCA, we performed GO and KEGG pathway analysis. In DEmRNA-related GO and KEGG terms, we mainly concerned the unique BP terms and pathway of different age groups. In group aged ≤ 39 years, the unique BP terms mainly included endothelial cell activation, mitotic cytokinetic process, and hyaluronan metabolic process. It should be noted that we have not enriched the unique KEGG pathway in this group, and may be related with case number and the cutoff criteria of DEmRNA. For patients aged 40–64 years, the unique BP terms were mainly related to cellular response to tumor necrosis factor, protein kinase A signaling, peptidyl-tyrosine phosphorylation, and regulation of phosphatidylinositol 3-kinase activity; the unique KEGG pathway were mainly cytokine-cytokine receptor interaction. In group aged ≥ 65 years, the unique BP terms mainly included negative regulation of metalloendopeptidase activity, extracellular regulation of signal transduction, primary amino compound metabolic process; the unique KEGG pathways included TGF-beta signaling pathway, TNF signaling pathway, and MicroRNAs in cancer. These enrichment results might support that age adds pathogenesis complexity in the BRCA, and our ceRNA network reflects special mechanisms in different age BRCA. These unique mechanisms worthy further to be concerned in drug development and refine therapeutic strategies.

In present study, we also performed the overall survival analysis, univariate and multiple Cox regression analysis of RNAs in ceRNA network. For patients aged ≤ 39 years, AGAP11 is not only associated with overall survival time, but also an independent prognostic factor. To date, no literature has shown the link between AGAP11 and cancer survival. For patients aged 40–64 years, there were five lncRNAs (ADAMTS9-AS1, LINC00461, LINC00536, SYNPR-AS1, and MME-AS1), and two miRNAs (has-mir-137 and has-mir-301b) identified to be associated with the overall survival. Among the seven overall survival related RNAs, has-mir-301b was identified as an independent prognostic factor. ADAMTS9-AS1, LINC00536, LINC00461, has-mir-137, and has-mir-301b have been reported to be associated with development and prognosis of BRCA (Ying et al., 2017; Dong et al., 2019). However, in this study, we found that these five RNAs were only associated with the prognosis of BRCA aged 40–64 years, which demonstrated the difference of the pathogenesis and prognosis in different age BRCA. Wang X. W. et al. (2019) identified that SYNPR-AS1 is overexpressed in female patients and adenocarcinoma of non-small cell lung cancer. One study reported that MME-AS1 was relative with prognosis in patients with intrahepatic cholangiocarcinoma (Kang et al., 2020). For the first time, we found that has-mir-301b played significant roles in the prognosis of patients aged 40–64 years, therefore, we suggest that new research can be carried out around it.

In addition, seven lncRNAs (AC027288.1, AC073342.2, AC110491.1, AC128709.1, ADAMTS9-AS1, AL021395.1, and TBL1XR1-AS1), two mRNAs (KIT and OSR1), and one miRNA (hsa-mir-383) were identified as prognostic indicators concerned with the overall survival in BRCA patients aged ≥ 65 years. To our knowledge, there is no research to clearly explain the function of AC027288.1, AC073342.2, AC128709.1, AL021395.1, and TBL1XR1-AS1 so far. ADAMTS9-AS1, KIT, OSR1, and hsa-mir-383 have been reported to be associated with the prognosis of BRCA (Kashiwagi et al., 2013; Li et al., 2020; Zhang J. et al., 2020). It is notable that KIT, OSR1, and hsa-mir-383 are merely associated with overall survival time in BRCA patients aged ≥ 65 years in present study. More interestingly, OSR1 is also an independent prognostic factor for patients aged ≥ 65 years. AC110491.1 has been reported to be linked with the prognosis of endometrial carcinoma and bladder cancer (Ouyang et al., 2019; Xu et al., 2019). For the first time, we found that AC110491.1 is associated with overall survival time of BRCA patients aged ≥ 65 years.

There are several limitations in present study. The number of patients aged ≤ 39 years available in TCGA database was relatively small, and thus more datasets are required for further validation. In addition, the findings of the present study should be verified and extended in larger studies, which will be conducted in our further studies. In summary, we identified the differences in the ceRNA regulatory networks for different age female BRCA. These unique RNAs might have clinical value for the diagnosis, refine therapeutic strategies and prognosis prediction in different age female BRCA patients. The results of the current study might be the foundation for future basic and clinical research. In addition, it is worth mentioning that several RNAs related to the diagnosis and prognosis were reported by multiple literatures are special for BRCA patients in certain age group, which indicated the importance of stratifying analysis and personalized medicine for BRCA patients according to age.

## Data Availability Statement

The original contributions presented in the study are included in the article/Supplementary Material, further inquiries can be directed to the corresponding author/s.

## Author Contributions

Q-TC and DQ-L conceived and designed the study. Z-QL and G-TZ performed the analysis procedures and drafted the manuscript. LJ and C-QL analyzed the results. All authors reviewed and approved the final version.

## Conflict of Interest

The authors declare that the research was conducted in the absence of any commercial or financial relationships that could be construed as a potential conflict of interest.

## Footnotes

1 https://portal.gdc.cancer.gov

2 http://www.mircode.org

3 http://www.targetscan.org/

4 http://www.mirdb.org/miRDB/

5 http://mirtarbase.mbc.nctu.edu.tw

## References

[B1] An Y. Zhang J. Cheng X. Li B. Tian Y. Zhang X. (2020). miR-454 suppresses the proliferation and invasion of ovarian cancer by targeting E2F6. *Cancer Cell Int.* 20:237. 10.1186/s12935-020-01300-0 32536825 PMC7291497

[B2] Brandt J. Garne J. P. Tengrup I. Manjer J. (2015). Age at diagnosis in relation to survival following breast cancer: a cohort study. *World J. Surg. Oncol.* 13:33. 10.1186/s12957-014-0429-x 25889186 PMC4344734

[B3] Chiang C. H. Chu P. Hou M. F. Hung W. C. (2016). MiR-182 promotes proliferation and invasion and elevates the HIF-1α-VEGF-A axis in breast cancer cells by targeting FBXW7. *Am. J. Cancer Res.* 6 1785–1798. 27648365 PMC5004079

[B4] Dong L. Qian J. Chen F. Fan Y. Long J. (2019). LINC00461 promotes cell migration and invasion in breast cancer through miR-30a-5p/integrin beta3 axis. *J. Cell. Biochem.* 120 4851–4862. 10.1002/jcb.27435 30623482

[B5] El Chediak A. Alameddine R. S. Hakim A. Hilal L. Abdel Massih S. Hamieh L. (2017). Younger age is an independent predictor of worse prognosis among Lebanese nonmetastatic breast cancer patients: analysis of a prospective cohort. *Breast Cancer (Dove Med. Press)* 9 407–414. 10.2147/BCTT.S130273 28670139 PMC5479304

[B6] Ergun S. Oztuzcu S. (2015). Oncocers ceRNA-mediated cross-talk by sponging miRNAs in oncogenic pathways. *Tumour Biol.* 36 3129–3136. 10.1007/s13277-015-3346-x 25809705

[B7] Fan C. N. Ma L. Liu N. (2018). Systematic analysis of lncRNA-miRNA-mRNA competing endogenous RNA network identifies four-lncRNA signature as a prognostic biomarker for breast cancer. *J. Transl. Med.* 16:264. 10.1186/s12967-018-1640-2 30261893 PMC6161429

[B8] Fu Y. Shao Z. He Q. Z. Jiang B. Q. Wu Y. Zhuang Z. G. (2015). Hsa-miR-206 represses the proliferation and invasion of breast cancer cells by targeting Cx43. *Eur. Rev. Med. Pharmacol. Sci.* 19 2091–2104. 26125274

[B9] Guo C. Shi H. Shang Y. Zhang Y. Cui J. Yu H. (2020). LncRNA LINC00261 overexpression suppresses the growth and metastasis of lung cancer via regulating miR-1269a/FOXO1 axis. *Cancer Cell Int.* 20:275. 10.1186/s12935-020-01332-6 32607060 PMC7318380

[B10] Guo G. Dai S. Chen Q. (2020). Long noncoding RNA LINC00261 reduces proliferation and migration of breast cancer cells via the NME1-EMT pathway. *Cancer Manag. Res.* 12 3081–3089. 10.2147/CMAR.S237197 32440206 PMC7210026

[B11] Kan Z. Ding Y. Kim J. Jung H. H. Chung W. Lal S. (2018). Multi-omics profiling of younger Asian breast cancers reveals distinctive molecular signatures. *Nat. Commun.* 9:1725. 10.1038/s41467-018-04129-4 29713003 PMC5928087

[B12] Kang Z. Guo L. Zhu Z. Qu R. (2020). Identification of prognostic factors for intrahepatic cholangiocarcinoma using long non-coding RNAs-associated ceRNA network. *Cancer Cell Int.* 20:315. 10.1186/s12935-020-01388-4 32694937 PMC7364620

[B13] Kashiwagi S. Yashiro M. Takashima T. Aomatsu N. Kawajiri H. Ogawa Y. (2013). c-Kit expression as a prognostic molecular marker in patients with basal-like breast cancer. *Br. J. Surg.* 100 490–496. 10.1002/bjs.9021 23319435

[B14] Li W. Jin X. Zhang Q. Zhang G. Deng X. Ma L. (2014). Decreased expression of miR-204 is associated with poor prognosis in patients with breast cancer. *Int. J. Clin. Exp. Pathol.* 7 3287–3292. 25031750 PMC4097245

[B15] Li Y. Jiao Y. Fu Z. Luo Z. Su J. Li Y. (2019). High miR-454-3p expression predicts poor prognosis in hepatocellular carcinoma. *Cancer Manag. Res.* 11 2795–2802. 10.2147/CMAR.S196655 31114333 PMC6497481

[B16] Li Y. Qin J. Wu J. Dai X. Xu J. (2020). High expression of OSR1 as a predictive biomarker for poor prognosis and lymph node metastasis in breast cancer. *Breast Cancer Res. Treat.* 182 35–46. 10.1007/s10549-020-05671-w 32424721

[B17] Liu H. Ye T. Yang X. Lv P. Wu X. Zhou H. (2020). A panel of four-lncRNA signature as a potential biomarker for predicting survival in clear cell renal cell carcinoma. *J. Cancer* 11 4274–4283. 10.7150/jca.40421 32368310 PMC7196268

[B18] Long J. Ou C. Xia H. Zhu Y. Liu D. (2015). MiR-503 inhibited cell proliferation of human breast cancer cells by suppressing CCND1 expression. *Tumour Biol.* 36 8697–8702. 10.1007/s13277-015-3623-8 26047605

[B19] Luo L. H. Jin M. Wang L. Q. Xu G. J. Lin Z. Y. Yu D. D. (2020). Long noncoding RNA TCL6 binds to miR-106a-5p to regulate hepatocellular carcinoma cells through PI3K/AKT signaling pathway. *J. Cell. Physiol.* 235 6154–6166. 10.1002/jcp.29544 32020591

[B20] Ma D. Jiang Y. Z. Xiao Y. Xie M. D. Zhao S. Jin X. (2020). Integrated molecular profiling of young and elderly patients with triple-negative breast cancer indicates different biological bases and clinical management strategies. *Cancer* 126 3209–3218. 10.1002/cncr.32922 32383785

[B21] Marino A. L. Evangelista A. Vieira R. A. Macedo T. Kerr L. M. Abrahão-Machado L. F. (2014). MicroRNA expression as risk biomarker of breast cancer metastasis a pilot retrospective case-cohort study. *BMC Cancer* 14:739. 10.1186/1471-2407-14-739 25277099 PMC4195914

[B22] Naorem L. D. Prakash V. S. Muthaiyan M. Venkatesan A. (2020). Comprehensive analysis of dysregulated lncRNAs and their competing endogenous RNA network in triple-negative breast cancer. *Int. J. Biol. Macromol.* 145 429–436. 10.1016/j.ijbiomac.2019.12.196 31883894

[B23] Ouyang D. Li R. Li Y. Zhu X. (2019). A 7-lncRNA signature predict prognosis of Uterine corpus endometrial carcinoma. *J. Cell. Biochem.* 120 18465–18477. 10.1002/jcb.29164 31168849

[B24] Qi L. Zhang T. Yao Y. Zhuang J. Liu C. Liu R. (2019). Identification of lncRNAs associated with lung squamous cell carcinoma prognosis in the competitive endogenous RNA network. *Peer J* 7:e7727. 10.7717/peerj.7727 31576252 PMC6753923

[B25] Qi X. Zhang D. H. Wu N. Xiao J. H. Wang X. Ma W. (2015). ceRNA in cancer: possible functions and clinical implications. *J. Med. Genet.* 52 710–718. 10.1136/jmedgenet-2015-103334 26358722

[B26] Ren L. Chen H. Song J. Chen X. Lin C. Zhang X. (2019). MiR-454-3p-mediated Wnt/beta-catenin signaling antagonists suppression promotes breast cancer metastasis. *Theranostics* 9 449–465. 10.7150/thno.29055 30809286 PMC6376193

[B27] Salmena L. Poliseno L. Tay Y. Kats L. Pandolfi P. P. (2011). A ceRNA hypothesis: the Rosetta Stone of a hidden RNA language? *Cell* 146 353–358. 10.1016/j.cell.2011.07.014 21802130 PMC3235919

[B28] Samaeekia R. Adorno-Cruz V. Bockhorn J. Chang Y. F. Huang S. Prat A. (2017). miR-206 inhibits stemness and metastasis of breast cancer by targeting MKL1/IL11 pathway. *Clin. Cancer Res.* 23 1091–1103. 10.1158/1078-0432.CCR-16-0943 27435395 PMC5247402

[B29] Shen S. Q. Huang L. S. Xiao X. L. Zhu X. F. Xiong D. D. Cao X. M. (2017). miR-204 regulates the biological behavior of breast cancer MCF-7 cells by directly targeting FOXA1. *Oncol. Rep.* 38 368–376. 10.3892/or.2017.5644 28534958

[B30] Song Y. Guo Q. Gao S. Hua K. (2019). miR-454-3p promotes proliferation and induces apoptosis in human cervical cancer cells by targeting TRIM3. *Biochem. Biophys. Res. Commun.* 516 872–879. 10.1016/j.bbrc.2019.06.126 31270028

[B31] Wang B. Wang H. Yang Z. (2012). MiR-122 inhibits cell proliferation and tumorigenesis of breast cancer by targeting IGF1R. *PLoS One* 7:e47053. 10.1371/journal.pone.0047053 23056576 PMC3466252

[B32] Wang J. Tsouko E. Jonsson P. Bergh J. Hartman J. Aydogdu E. (2014). miR-206 inhibits cell migration through direct targeting of the actin-binding protein coronin 1C in triple-negative breast cancer. *Mol. Oncol.* 8 1690–1702. 10.1016/j.molonc.2014.07.006 25074552 PMC5528580

[B33] Wang K. Liao C. Zhong Q. Dong H. Zhang T. Jin R. (2019). CeNETs analysis reveals the prognostic value of a signature integration from five lncRNAs in breast cancer. *J. Cell. Biochem.* 120 13509–13519. 10.1002/jcb.28626 30927387

[B34] Wang X. Gao X. Tian J. Zhang R. Qiao Y. Hua X. (2020). LINC00261 inhibits progression of pancreatic cancer by down-regulating miR-23a-3p. *Arch. Biochem. Biophys.* 689:108469. 10.1016/j.abb.2020.108469 32590069

[B35] Wang X. W. Guo Q. Q. Wei Y. Ren K. M. Zheng F. S. Tang J. (2019). Construction of a competing endogenous RNA network using differentially expressed lncRNAs, miRNAs and mRNAs in nonsmall cell lung cancer. *Oncol. Rep.* 42 2402–2415. 10.3892/or.2019.7378 31638248 PMC6859443

[B36] Wang Z. Wang X. (2020). miR-122-5p promotes aggression and epithelial-mesenchymal transition in triple-negative breast cancer by suppressing charged multivesicular body protein 3 through mitogen-activated protein kinase signaling. *J. Cell. Physiol.* 235 2825–2835. 10.1002/jcp.29188 31541468

[B37] Xu Z. Wang C. Xiang X. Li J. Huang J. (2019). Characterization of mRNA expression and endogenous RNA profiles in bladder cancer based on the cancer genome atlas (TCGA) database. *Med. Sci. Monit.* 25 3041–3060. 10.12659/MSM.915487 31020952 PMC6498884

[B38] Yan J. Xu Y. Wang H. Du T. Chen H. (2017). MicroRNA-503 inhibits the proliferation and invasion of breast cancer cells via targeting insulin-like growth factor 1 receptor. *Mol. Med. Rep.* 16 1707–1714. 10.3892/mmr.2017.6816 28656281 PMC5562074

[B39] Ying X. Sun Y. He P. (2017). MicroRNA-137 inhibits BMP7 to enhance the epithelial-mesenchymal transition of breast cancer cells. *Oncotarget* 8 18348–18358. 28407692 10.18632/oncotarget.15442 PMC5392333

[B40] Zhang J. Kong X. Shi Q. Zhao B. (2020). MicroRNA-383-5p acts as a potential prognostic biomarker and an inhibitor of tumor cell proliferation, migration, and invasion in breast cancer. *Cancer Biomark.* 27 423–432. 10.3233/CBM-190704 31903982 PMC12662315

[B41] Zhang S. Cao R. Li Q. Yao M. Chen Y. Zhou H. (2019). Comprehensive analysis of lncRNA-associated competing endogenous RNA network in tongue squamous cell carcinoma. *Peer J* 7:e6397. 10.7717/peerj.6397 30755833 PMC6368841

[B42] Zhang Y. Li Z. Chen M. Chen H. Zhong Q. Liang L. (2020). lncRNA TCL6 correlates with immune cell infiltration and indicates worse survival in breast cancer. *Breast Cancer* 27 573–585. 10.1007/s12282-020-01048-5 31960363

[B43] Zhao Z. Fan X. Jiang L. Xu Z. Xue L. Zhan Q. (2017). miR-503-3p promotes epithelial-mesenchymal transition in breast cancer by directly targeting SMAD2 and E-cadherin. *J. Genet. Genomics* 44 75–84. 10.1016/j.jgg.2016.10.005 28161325

[B44] Zuo J. Yu H. Xie P. Liu W. Wang K. Ni H. (2019). miR-454-3p exerts tumor-suppressive functions by down-regulation of NFATc2 in glioblastoma. *Gene* 710 233–239. 10.1016/j.gene.2019.06.008 31181312

