# Supplementary material for: Construction and Comparison of ceRNA Regulatory Network for Different Age Female Breast Cancer
Source: Front Genet. 2021 Apr 21;12:603544. doi: 10.3389/fgene.2021.603544 (PMC8097183; doi:10.3389/fgene.2021.603544)
Supplement: Supplementary file 1 [file Data_Sheet_1.docx]

**SUPPLMENTARY TABLES**

**SUPPLMENT TABLE 1** The unique DEmRNAs involved in the ceRNA networks of different age BRCA patients

| **Group** | **mRNA** | **Log_2_FC** | ***P* Value** | **FDR** |
| --- | --- | --- | --- | --- |
| age ≤39 | HOXA9 | -2.30390476 | 2.27E-07 | 4.75E-06 |
|  | DEPDC1 | 3.042276493 | 6.24E-10 | 2.38E-08 |
|  | CEP55 | 3.23455332 | 2.13E-12 | 1.4E-10 |
| age 40-64 | TTN | -5.43389 | 4.84E-266 | 3.13E-263 |
|  | CCL1 | -3.13425 | 3.80E-85 | 1.68E-83 |
|  | KLHL40 | -7.32334 | 1.99E-92 | 9.93E-91 |
|  | SFRP1 | -2.36219 | 2.27E-28 | 1.97E-27 |
| age ≥ 65 | DDC | 2.341175 | 0.002693 | 0.004955 |
|  | JPH1 | 2.11271 | 7.91E-09 | 3.47E-08 |
|  | NPTX1 | 2.668447 | 0.000345 | 0.000741 |
|  | GREM2 | -2.35427 | 2.04E-22 | 3.62E-21 |
|  | ANGPTL2 | -2.10586 | 1.29E-42 | 6.79E-41 |
|  | RECK | -2.08801 | 2.8E-46 | 1.66E-44 |
|  | CREB5 | -2.38688 | 4.46E-43 | 2.4E-41 |
|  | HOXB5 | 2.188422 | 2.06E-05 | 5.47E-05 |

**SUPPLMENT TABLE 2** The unique DEmiRNAs involved in the ceRNA networks of different age BRCA patients

| **Group** | **miRNA** | **Log_2_FC** | ***P* Value** | **FDR** |
| --- | --- | --- | --- | --- |
| age ≤39 | hsa-mir-100 | -2.403520152 | 3.32E-18 | 4.75E-16 |
|  | hsa-mir-454 | 2.254695048 | 7.52E-06 | 0.000122887 |
| age 40-64 | hsa-mir-206 | -4.07935808 | 5.45E-19 | 3.06E-18 |
|  | hsa-mir-122 | 3.313593991 | 4.42E-07 | 9.64E-07 |
| age ≥ 65 | hsa-mir-503 | 2.046451389 | 1.66E-10 | 1.17E-09 |

**SUPPLMENT TABLE 3** The unique DElncRNAs involved in the ceRNA networks of different age BRCA patients

| **Group** | **lncRNA** | **Log_2_FC** | ***P* Value** | **FDR** |
| --- | --- | --- | --- | --- |
| age39 | WARS2-IT1 | -2.121553989 | 1.22052E-08 | 8.64839E-07 |
|  | JARID2-AS1 | 2.1290781 | 0.000226845 | 0.003228023 |
|  | C9orf106 | 2.036142706 | 0.000939149 | 0.009485877 |
|  | SPIN4-AS1 | -2.054538907 | 2.20227E-05 | 0.00051174 |
|  | AC006487.1 | -2.123116568 | 8.66213E-06 | 0.000225383 |
|  | GRIK1-AS1 | -2.154144029 | 9.88956E-05 | 0.001691746 |
|  | FAM181A-AS1 | -2.16502602 | 0.000157664 | 0.002415025 |
|  | MIR210HG | 2.112109985 | 0.000102859 | 0.001748377 |
| age 40-64 | LINC00261 | 5.64994283 | 7.67034E-07 | 2.32067E-06 |
|  | E2F3-IT1 | 2.10465196 | 1.57625E-05 | 3.92657E-05 |
|  | ARHGEF3-AS1 | 2.011378268 | 0.000102766 | 0.000225565 |
|  | RMRP | 5.26237833 | 5.12861E-08 | 1.86044E-07 |
|  | KIRREL3-AS1 | 2.350191755 | 2.27058E-05 | 5.52585E-05 |
|  | LINC00494 | 2.319394024 | 1.51435E-13 | 1.04401E-12 |
|  | AC025211.1 | 2.182323616 | 0.000130419 | 0.000281201 |
|  | AL354984.1 | 2.185256801 | 1.10789E-07 | 3.82503E-07 |
|  | C21orf91-OT1 | 2.24425226 | 1.03618E-05 | 2.6459E-05 |
|  | AL589642.1 | 4.164822044 | 1.72641E-07 | 5.77404E-07 |
|  | AC138761.1 | 3.315055436 | 2.51963E-07 | 8.23613E-07 |
|  | CACNA1C-IT3 | 2.060683613 | 0.000301429 | 0.000613187 |
|  | UCA1 | 2.610587962 | 3.35484E-12 | 2.04E-11 |
|  | LINC00404 | 3.439214092 | 1.48856E-05 | 3.72206E-05 |
|  | DLX6-AS1 | 2.185990812 | 2.90361E-08 | 1.08936E-07 |
|  | LINC00442 | 2.750267903 | 6.74579E-05 | 0.000152511 |
|  | TCL6 | 3.483081346 | 2.36885E-18 | 2.56719E-17 |
|  | LINC00488 | 2.950231446 | 1.87652E-06 | 5.35381E-06 |
|  | C17orf102 | 2.815998271 | 5.13688E-07 | 1.59714E-06 |
|  | TLR8-AS1 | 2.240887718 | 1.81518E-07 | 6.05225E-07 |
|  | AL139002.1 | 3.523033248 | 1.13189E-05 | 2.87335E-05 |
|  | MAST4-IT1 | 2.479990313 | 7.6928E-06 | 2.00759E-05 |
|  | DSCR4 | 4.28517654 | 3.31206E-07 | 1.06219E-06 |
|  | NAALADL2-AS2 | 2.056266093 | 1.15437E-07 | 3.97284E-07 |
|  | LINC00113 | 2.576442639 | 1.77524E-07 | 5.92818E-07 |
|  | LINC00355 | 3.699686385 | 2.25258E-07 | 7.40511E-07 |
|  | OPCML-IT1 | 4.263072574 | 5.96894E-06 | 1.58543E-05 |
|  | AL356310.1 | 2.815003719 | 7.13076E-18 | 7.34188E-17 |
|  | ST7-AS2 | 2.412697208 | 4.6493E-07 | 1.46181E-06 |
|  | FNDC1-IT1 | 2.684427754 | 7.57345E-17 | 7.20643E-16 |
| 12 | FAM155A-IT1 | 2.318837065 | 9.73303E-06 | 2.49787E-05 |
|  | C12orf77 | 2.092423552 | 4.45513E-08 | 1.62978E-07 |

**SUPPLMENT TABLE 3** The unique DElncRNAs involved in the ceRNA networks of different age BRCA patients （continued）

| **Group** | **lncRNA** | **Log_2_FC** | ***P* Value** | **FDR** |
| --- | --- | --- | --- | --- |
| age ≥65 | PEX5L-AS1 | 2.965410275 | 6.6843E-05 | 0.000267435 |
|  | ADARB2-AS1 | 5.135383852 | 0.000270443 | 0.000912973 |
|  | AL021395.1 | 2.527404602 | 0.000137793 | 0.000505461 |
|  | TBL1XR1-AS1 | 2.007122273 | 5.1041E-05 | 0.000209761 |
|  | AC073263.1 | 2.015358945 | 3.56551E-06 | 1.91745E-05 |
|  | AC063962.1 | 2.577946994 | 7.20808E-07 | 4.49245E-06 |
|  | DIO3OS | -2.407126731 | 2.74673E-25 | 1.32941E-23 |
|  | LINC00210 | 5.422655686 | 0.000634557 | 0.001923345 |
|  | AC073342.2 | 2.592456879 | 0.00036052 | 0.001179452 |
|  | HPYR1 | 3.040976931 | 1.77868E-07 | 1.26282E-06 |
|  | OXCT1-AS1 | -2.308345153 | 3.70125E-42 | 5.13927E-40 |
|  | C5orf17 | 2.592644008 | 0.000923445 | 0.002663117 |
|  | AC027288.1 | 2.49396294 | 0.000287591 | 0.000965092 |
|  | AC110491.1 | -2.536043322 | 2.64767E-12 | 4.20747E-11 |
|  | AL589765.1 | 2.050764065 | 1.21052E-05 | 5.75369E-05 |
|  | LINC00243 | 2.796876501 | 3.08173E-13 | 5.42667E-12 |
|  | AC128709.1 | 2.081201826 | 3.21234E-05 | 0.000139318 |
|  | SHANK2-AS3 | 2.858682607 | 0.000579693 | 0.001782862 |
|  | C9orf163 | 2.34741282 | 5.42931E-23 | 2.23234E-21 |
|  | AC104472.1 | 2.43996668 | 2.07709E-05 | 9.40297E-05 |
|  | AP002478.1 | 2.033917008 | 0.001784345 | 0.004834743 |
|  | LINC00351 | 3.649311458 | 0.002203585 | 0.005765945 |
|  | AC007431.1 | 2.536894437 | 6.5343E-06 | 3.30618E-05 |
|  | AC005544.1 | 2.848326297 | 3.04268E-10 | 3.63491E-09 |
|  | AC112721.1 | 4.028404422 | 4.64452E-15 | 1.03524E-13 |
|  | MACROD2-AS1 | 2.405480424 | 0.001234131 | 0.003468178 |
|  | COL4A2-AS2 | -2.260643023 | 2.13455E-11 | 2.94937E-10 |
|  | WASIR2 | 2.46510321 | 6.95752E-11 | 8.83511E-10 |
|  | HOTTIP | 2.561867852 | 0.00307354 | 0.007690661 |
|  | MAGI2-AS3 | -2.276268642 | 2.47027E-53 | 8.71801E-51 |
|  | ERVH48-1 | 2.233970206 | 2.58598E-05 | 0.000114497 |

**SUPPLMENT TABLE 4** clinicopathological characteristics of each age group patient.

| **Characteristics** | **≤39** | **40-64** | **≥ 65** |
| --- | --- | --- | --- |
| **Histological type** |  |  |  |
| Infiltrating Ductal Carcinoma | 67 | 400 | 177 |
| Infiltrating lobular Carcinoma | 0 | 43 | 25 |
| Other | 5 | 41 | 25 |
| **Clinical stage** |  |  |  |
| Stage Ⅰ | 13 | 86 | 42 |
| Stage Ⅱ | 38 | 290 | 125 |
| Stage Ⅲ | 21 | 97 | 56 |
| Stage Ⅳ | 0 | 11 | 4 |
| **Lymph node status** |  |  |  |
| Negative | 29 | 230 | 121 |
| Positive | 43 | 254 | 106 |
| **ER** |  |  |  |
| Negative | 18 | 118 | 42 |
| Positive | 54 | 366 | 185 |
| **PR** |  |  |  |
| Negative | 25 | 164 | 64 |
| Positive | 47 | 320 | 163 |
| **HER2** |  |  |  |
| Negative | 62 | 414 | 194 |
| Positive | 10 | 70 | 33 |
