# Supplementary material for: Construction and Comparison of ceRNA Regulatory Network for Different Age Female Breast Cancer
Source: Front Genet. 2021 Apr 21;12:603544. doi: 10.3389/fgene.2021.603544 (PMC8097183; doi:10.3389/fgene.2021.603544)
Supplement: Supplementary file 2 [file Data_Sheet_2.pdf]

Supplementary Figures

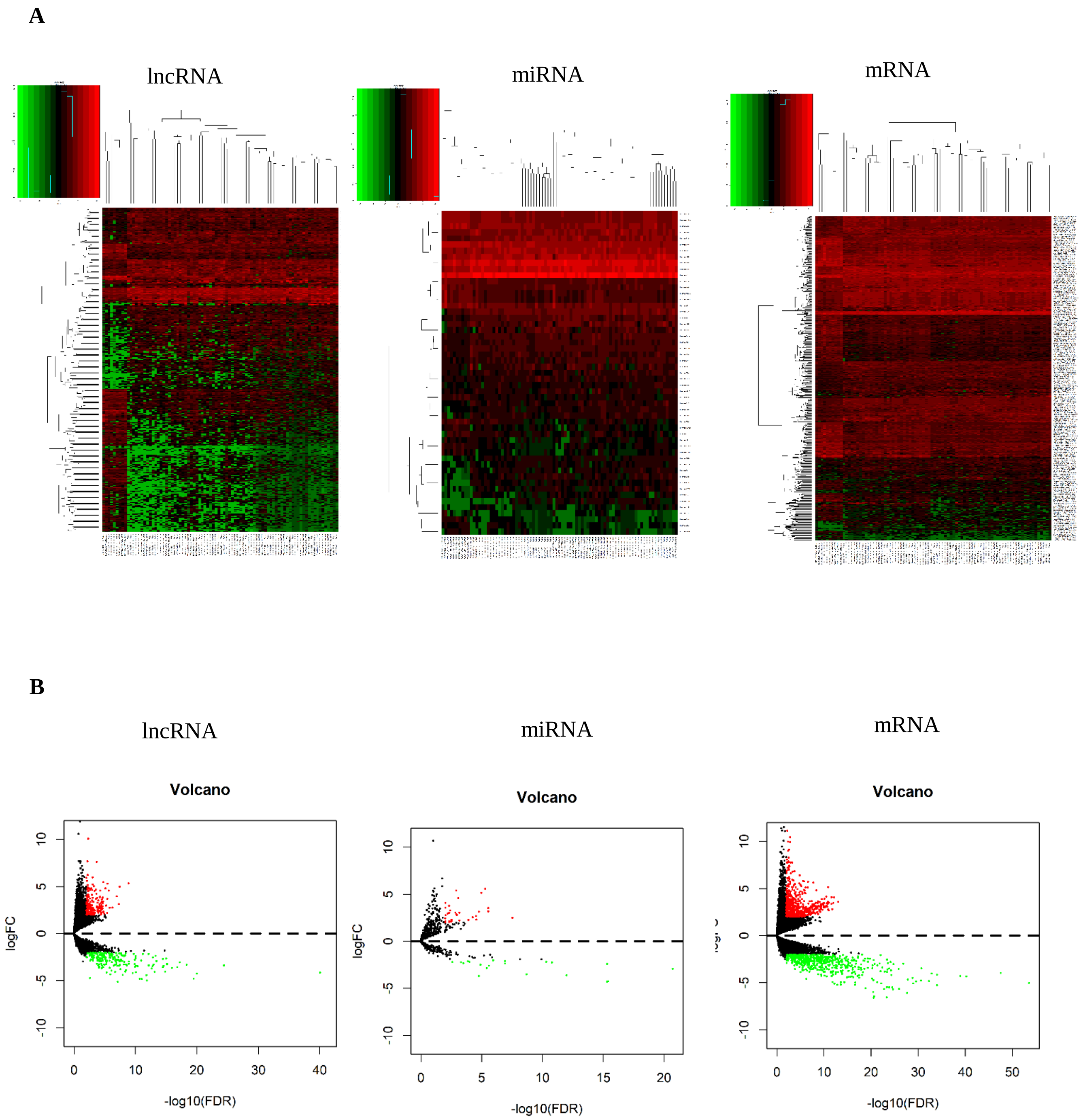

SUPPLMENTARY FIGURE S1 | Gene expression analysis of BRCA aged ≤39 years data from TCGA database. A, Heatmaps of differentially expressed RNAs between breast cancer and non-tumor tissues. B, Volcano plots of differentially expressed lncRNAs, miRNAs and mRNAs. The red points in the plots represent upregulated RNAs and the green points represent downregulated RNAs with statistical significance.

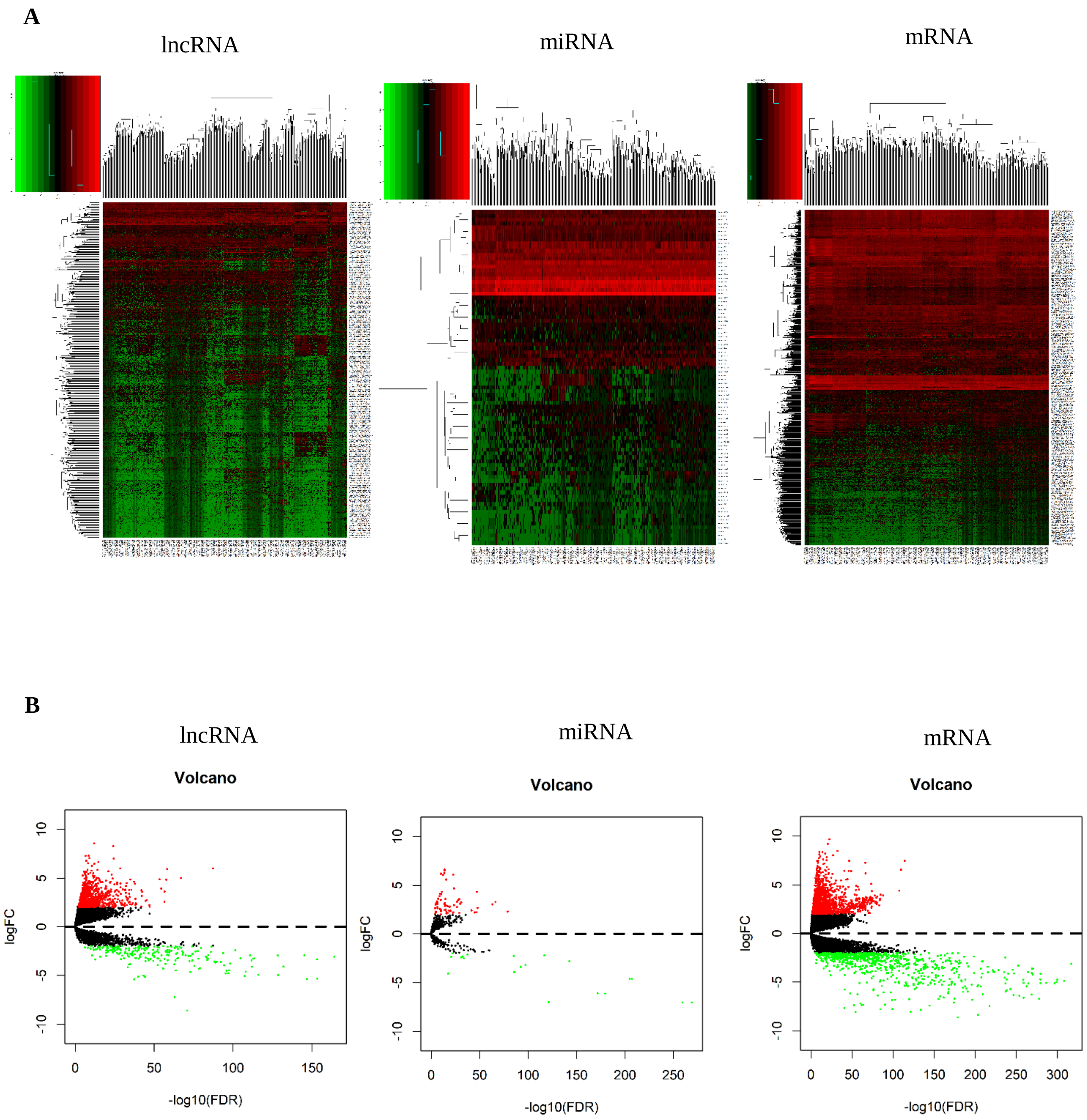

SUPPLMENTARY FIGURE S2 | Gene expression analysis of BRCA aged 40-64 years data from TCGA database. A, Heatmaps of differentially expressed RNAs between breast cancer and non-tumor tissues. B, Volcano plots of differentially expressed lncRNAs, miRNAs and mRNAs. The red points in the plots represent upregulated RNAs and the green points represent downregulated RNAs with statistical significance.

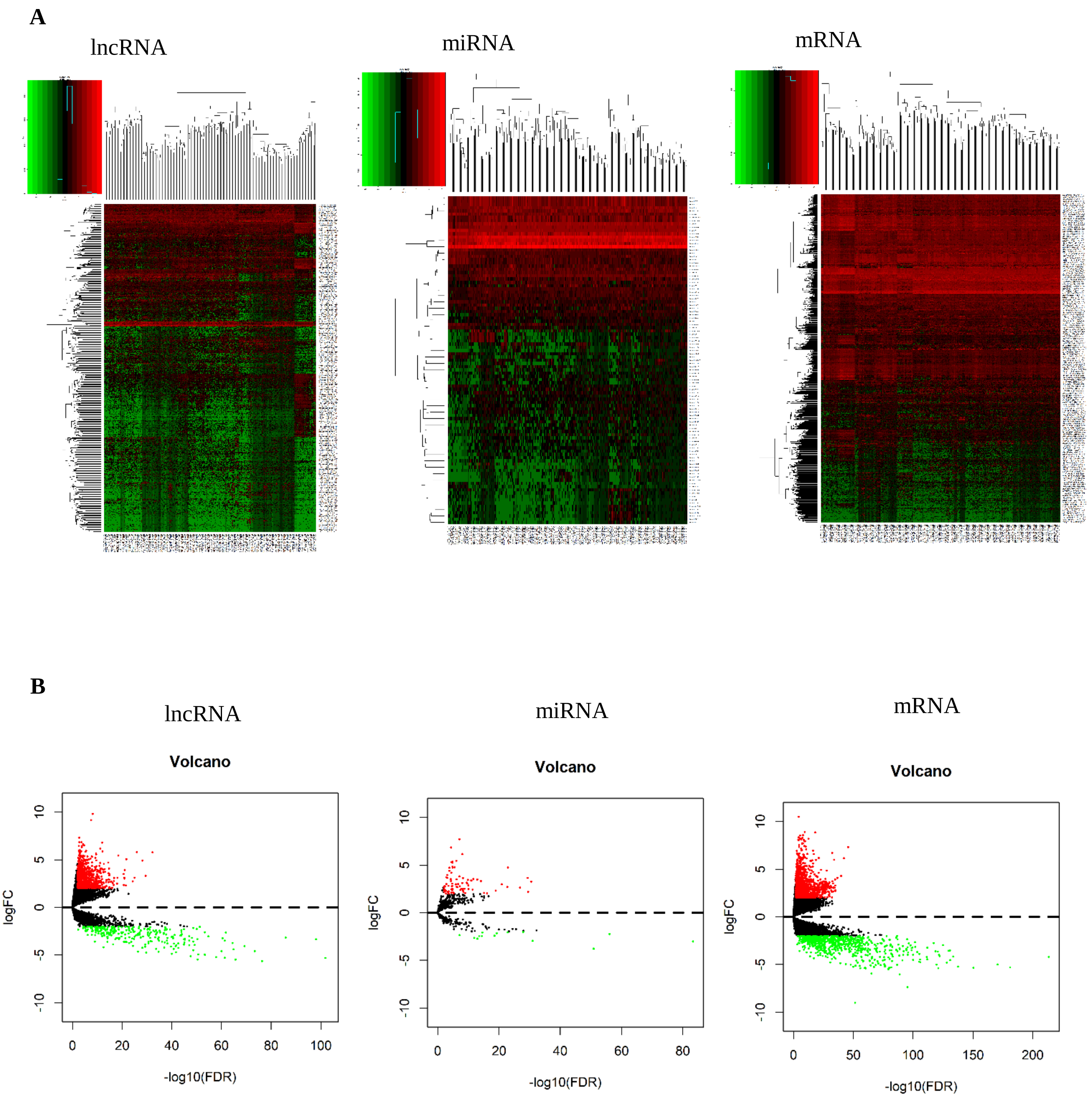

SUPPLMENTARY FIGURE S3 | Gene expression analysis of BRCA aged ≥65 years data from TCGA database. A, Heatmaps of differentially expressed RNAs between breast cancer and non-tumor tissues. B, Volcano plots of differentially expressed lncRNAs, miRNAs and mRNAs. The red points in the plots represent upregulated RNAs and the green points represent downregulated RNAs with statistical significance.

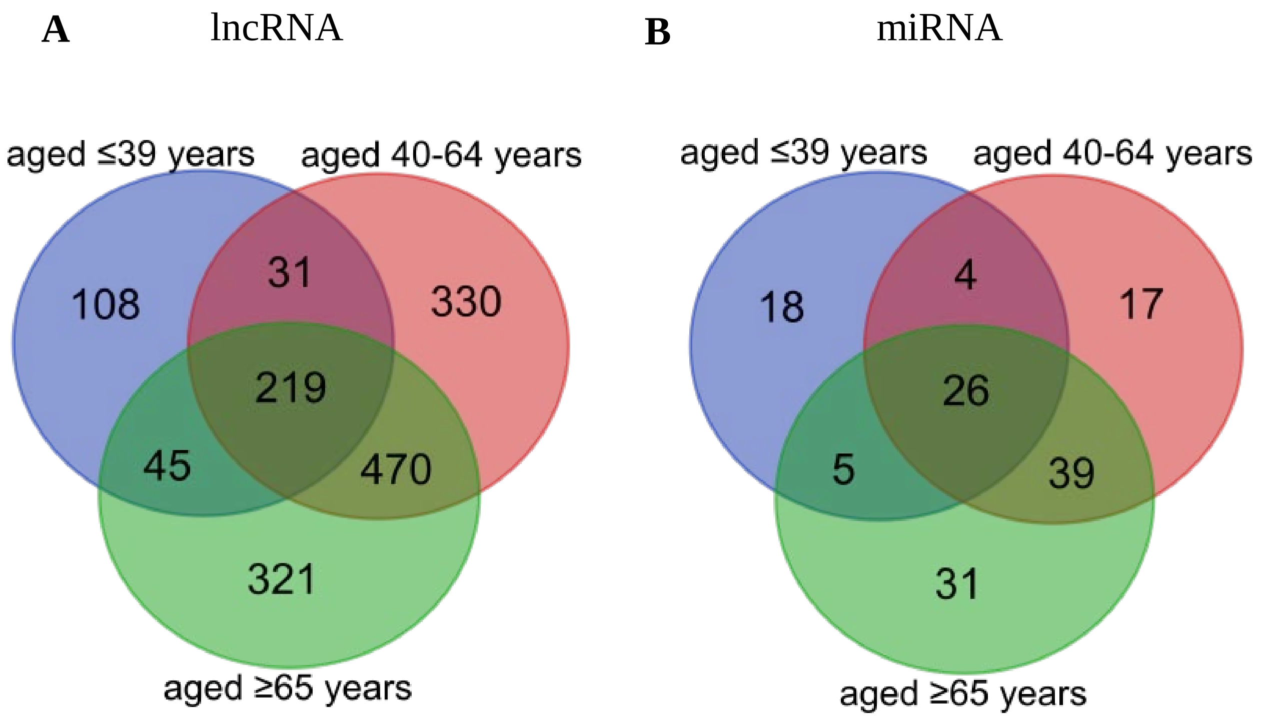

SUPPLMENTARY FIGURE S4 | Venn diagram of deregulated ncRNAs in each age group.

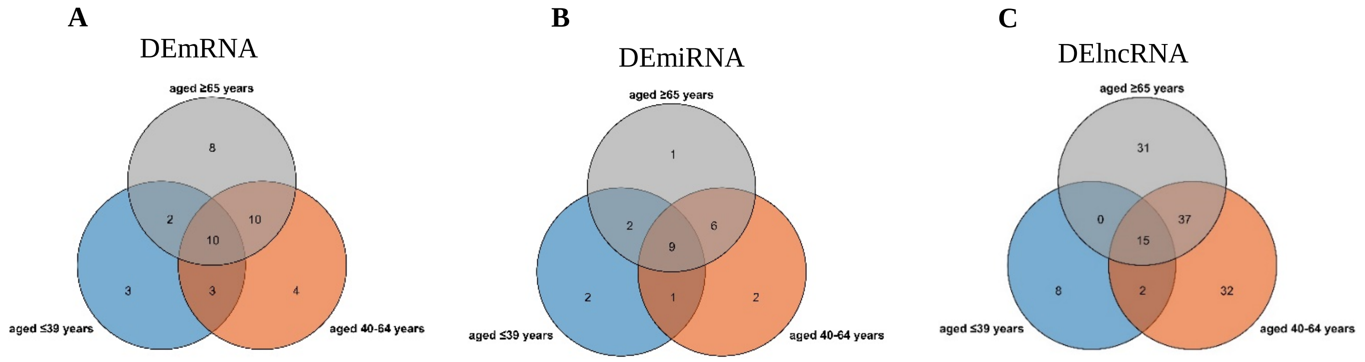

SUPPLMENTARY FIGURE S5 | Venn diagram of differentially expressed RNAs involved in the ceRNA network. A, DEmRNA; B, DEmiRNA; C, DElncRNA
